# Worsening calcification propensity precedes all-cause and cardiovascular mortality in haemodialyzed patients

**DOI:** 10.1038/s41598-017-12859-6

**Published:** 2017-10-17

**Authors:** Georg Lorenz, Dominik Steubl, Stephan Kemmner, Andreas Pasch, Wilhelm Koch-Sembdner, Dang Pham, Bernhard Haller, Quirin Bachmann, Christopher C. Mayer, Siegfried Wassertheurer, Susanne Angermann, Maciej Lech, Philipp Moog, Axel Bauer, Uwe Heemann, Christoph Schmaderer

**Affiliations:** 1 Department of nephrology, Klinikum rechts der Isar, Technical University Munich, Munich, Germany; 2 Calciscon AG, Biel-Nidau, Switzerland; 3 Institute of Medical Statistics and Epidemiology, Technical University Munich, Munich, Germany; 4 AIT Austrian Institute of Technology, Center for Health & Bioresources, Biomedical Systems, Vienna, Austria; 5 Department of cardiology Ludwig-Maximilian University, Munich, Germany

## Abstract

A novel *in-vitro* test (T_50_-test) assesses *ex-vivo* serum calcification propensity which predicts mortality in HD patients. The association of longitudinal changes of T_50_ with all-cause and cardiovascular mortality has not been investigated. We assessed T_50_ in paired sera collected at baseline and at 24 months in 188 prevalent European HD patients from the ISAR cohort, most of whom were Caucasians. Patients were followed for another 19 [interquartile range: 11–37] months. Serum T_50_ exhibited a significant decline between baseline and 24 months (246 ± 64 to 190 ± 68 minutes; p < 0.001). With serum Δ-phosphate showing the strongest independent association with declining T_50_ (r = −0.39; p < 0.001) in multivariable linear regression. The rate of decline of T_50_ over 24 months was a significant predictor of all-cause (HR = 1.51 per 1SD decline, 95% CI: 1.04 to 2.2; p = 0.03) and cardiovascular mortality (HR = 2.15; 95% CI: 1.15 to 3.97; p = 0.02) in Kaplan Meier and multivariable Cox-regression analysis, while cross-sectional T_50_ at inclusion and 24 months were not. Worsening serum calcification propensity was an independent predictor of mortality in this small cohort of prevalent HD patients. Prospective larger scaled studies are needed to assess the value of calcification propensity as a longitudinal parameter for risk stratification and monitoring of therapeutic interventions.

## Introduction

Disturbed calcium and phosphate homeostasis, vascular disease progression and excess mortality, largely attributable to cardiovascular (CV) causes, remain unresolved issues in haemodialysis (HD) patients^1–3^. Non-traditional risk factors, e.g. chronic inflammation, malnutrition and aberrant bone turn over gain increasing importance along with declining renal function^4^. Besides monitoring and targeting calcium and phosphate, the physiological humoral system, which resists the formation of calcium-phosphate-nanocrystal-formation in biological fluids, has largely been neglected.

Recently, Pasch *et al*. developed an *in-vitro* test (T_50_-test), that time-dependently assesses the calcification propensity of human serum in the presence of supersaturating doses of calcium and phosphate^5,6^. In this process, amorphous primary calciprotein particles (CPP) spontaneously grow into crystallized secondary CPP^5^. The transformation time T_50_ is thought to reflect the complex interplay of anti- and pro-calcifying serum components, such as calcium and phosphate on the one and such as magnesium, fetuin-A and albumin on the other hand^5–7^. A shorter T_50_ time was associated with accelerated vascular stiffness progression and overall mortality in chronic kidney disease stage III-IV^7^. Lower T_50_ values were also shown to predict graft failure, CV and all-cause mortality in renal transplant recipients^8,9^.

A recently published study revealed a moderate, yet independent association of decreased serum T50 with mortality and CV endpoints in patients of the original “Evaluation of Cinacalcet Therapy to Lower Cardiovascular Events” (EVOLVE) trial^10,11^. These data originate from 2785 HD patients with secondary hyperparathyroidism^10,11^. The moderate predictive capacity of T_50_ for all-cause mortality (HR = 1.1 per 1 SD lower T_50_) in the EVOLVE cohort was somewhat surprising considering that “pro-calcific pressure” should be particularly high in HD patients^10,12^. Interestingly, individual serum calcification propensity (T_50_-value) is to a large extent genetically determined^13^. This finding led us to hypothesize that it might not only be the absolute T_50_-value at a given time point, which mediated the risk of dialysis patients. Alternatively, changes to the individual’s resilience against CPP formation – a decline in T_50_ – throughout dialysis dependency might mirror risk more accurately. We therefore assessed T_50_ from serum samples collected at baseline and after 24 months in 188 HD patients to test if intra-individual changes in T_50_ precede mortality.

## Results

**Figure 1:**
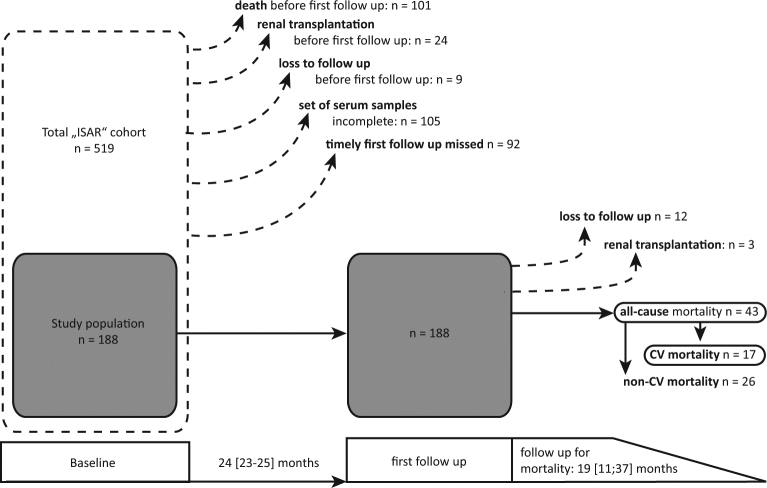
Observation schedule, endpoints and selection of the study population; Patients were selected per predefined criteria stated in “methods”. Dotted arrows indicate patients not selected or leaving the study. Sera were collected at inclusion and after 24 [23–25] months of observation. Patients were then followed up for mortality for an average of 19 [11; 37] months.

This study was performed in a subgroup of 188 European haemodialysis patients of the ISAR cohort who were still alive and active in the study after the first follow up (FU) period of 24 months with a maximum spread of ± 3.2 months and for whom sera were available at both baseline and FU. Patients for whom timely 24 [23–25] months FU had been missed were excluded a priori (Fig. 1). Most patients were of Caucasian ethnicity with only four black (2%) and two Chinese (1%) participants.

In comparison with the total cohort, the study population did not significantly differ with regard to demographic parameters, body mass index (BMI) and dialysis modality. Included patients were, by nature of the study design, enriched for survivors (22.9% versus 35.8% all-cause mortality in the total cohort; p < 0.001). Additionally, history of myocardial infarction (14.4% versus 19.8%; p = 0.02) and use of central venous catheters (2.7% versus 7.3%; p = 0.001) at baseline were less frequent among included patients. Median albumin levels were slightly higher in the study population (41 g/l versus 40 g/l, p = 0.04). For details please see supplementary Table 1.

### Serum calcification propensity declines in stable haemodialysis patients

**Figure 2:**
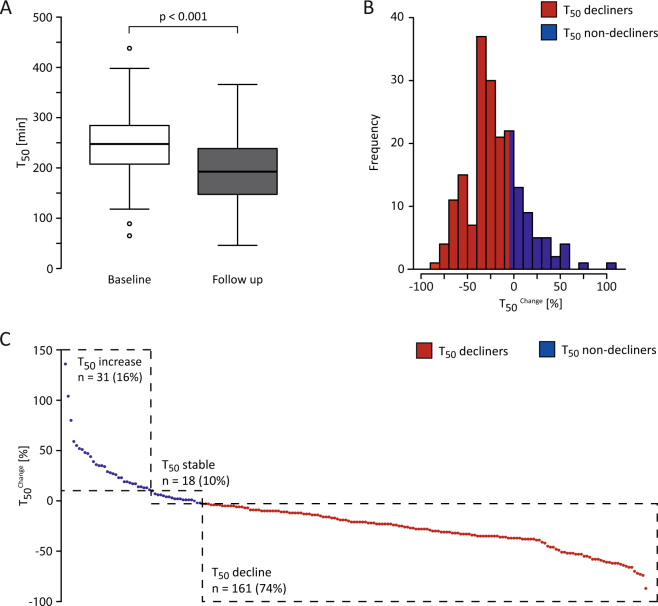
Absolute T_50_ at baseline and FU and percentage decline of T_50_; (**A**) Absolute T_50_ values at baseline (white boxplot) and at 24 months FU (grey boxplot). (**B**) Histogram stating the frequencies of T_50_^Change^ ((=T_50_^Follow up^ − T_50_^Baseline^)/(T_50_^Baseline^)) of n = 188 Patients. (**C**) Individual patients were sorted in descending order per their T_50_^Change^ values. Patients displaying a decline in T_50_ (defined by T_50_^Change^ < −5.1%; 5,1% was the inter-assay coefficient for T_50_ standards at 260 min in the EVOLVE cohort) were stained red. Absolute number of patients and (percent of total) are reported with dashed rectangles. The same colour code was used to stain the histogram in **B**.

Both T_50_^Baseline^ and T_50_^Follow up^ values were nearly normally distributed in this cohort (Supplementary Figure 1). Mean T_50_ values were 246 ± 64 minutes at baseline and 190 ± 68 minutes at time of 24 months FU indicating a significantly declining T_50_ over time (p < 0.001; Fig. 2A). In accordance, the histogram of T_50_^Change^ was skewed towards negative values (Fig. 2B). Of 188 patients included 31 (16%) presented with increasing T50 values. 18 (10%) patients showed stable values as defined by T_50_^Change^ = 0 ± 5,1% (5,1% was the inter-assay coefficient for T_50_ standards at 260 min in the EVOLVE cohort)^10^. Remarkably, a total of 106 (77%) patients presented declining T50 values (T_50_^Change^ < −5,1%) during 24 months of FU (Fig. 2B).

### Description of the study cohort and factors associated with declining T_50_ (low T_50_^Change^)

**Figure 3:**
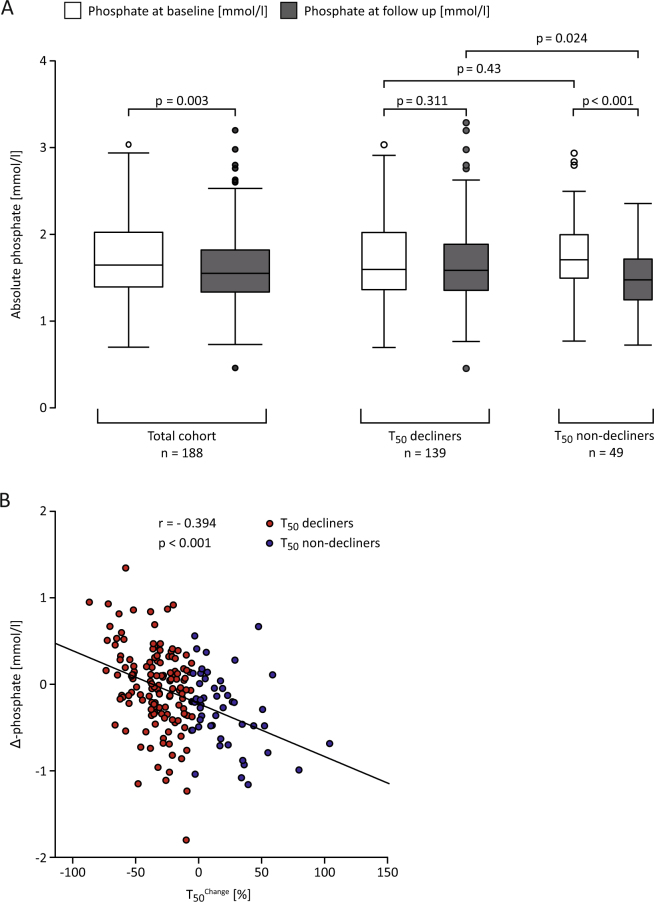
Relation of serum phosphate and T_50_^Change^; (**A**) Left: Absolute phosphate levels at baseline (broadest white boxplot) and FU (broadest grey box plot) in the total cohort (n = 188). This cohort was split per T_50_ decliners versus T_50_ non-decliners Middle: Absolute phosphate levels at baseline (white boxplot) and FU (grey boxplot) in those that declined in T_50_ between baseline and FU (T_50_^Change^  <  −5.1%); n = 139. Right: Absolute phosphate levels at baseline (small white boxplot) and FU (small grey boxplot) in those that had stable or increasing T_50_ (T_50_^Change^ ≥ −5.1%); n = 49. Differences among groups regarding absolute phosphate levels were tested using paired and unpaired t-test as appropriate. (**B**) Dot plot depicting the correlation of T_50_^Change^ with ∆ phosphate (=phosphate ^Follow up^−phosphate ^Baseline^). T_50_ decliners and non-decliners were coloured red and blue, respectively. Pearson correlation was used to quantify the relation.

Table 1 displays the characteristics of the study population stratified by median T_50_^Change^ (above or below −22.7%) or by median absolute T_50_^Follow up^ (above or below 192.5 min) at time of 24 months FU. Median age of the entire cohort was 71 [57–78] years. Comparing the subgroups generated by medians, no significant associations were detectable for T_50_^Change^ with age, pre-existing comorbidities (including the adapted CCI) and intake of phosphate lowering drugs. However, in univariate analysis, increased serum phosphate was higher in those with T_50_^Change^ below the median (1.7± 0.5 versus 1.5 ± 0.4 mmol/l, p < 0.001). Interestingly, Δ phosphate (=phosphate^Follow up^ – phosphate^Baseline^) was also distributed differently among individuals above and below the median of T_50_^Change^ (Table 1). Whereas in the total study population absolute phosphate levels tended to decline between baseline (1.7 ± 0.5 mmol/l) and FU (1.6 ± 0.5 mmol/l; p = 0.003), patients with declining T_50_ values (T_50_^Change^ < −5.1%) presented with stable high phosphate levels (1.7 ± 0.5 mmol/l; p^paired t-test^ = 0.31). Subjects presenting with stable or increasing T_50_ at baseline and 24 months FU showed declining phosphate levels between baseline and 24 months FU (1.8 versus 1.5 ± 0.4 mmol/l, respectively; p < 0.001). Both groups did not significantly differ in phosphate levels measured at baseline (p = 0.43) but exhibited significantly different phosphate at 24 months FU (Fig. 3A). Δ phosphate was significantly correlated with T_50_^Change^ (r = −0.39; p < 0.001; Fig. 3B), suggesting a permanently elevated or increasing phosphate associates with declining T_50_. Δ phosphate remained the strongest regressor of T_50_^Change^ in multivariable linear regression (β = −8.4; 95%CI: −13.1 to−3.6; p = 0.001). R² of the model created was 0.24 - “(enter method)”. Additionally, Kt/V was a significant regressor of T_50_^Change^ (β = 5.4; 95% CI: 0.8 to 9.9; p = 0.001; Table 2). Of note, lower absolute T50 at FU was associated with higher parathyroid hormone and IL-6 levels (p = 0.01 and 0.03 respectively, Table 1).

**Table 1:** Characteristics of the study population stratified by median change of T_50_ and median absolute T_50_ at time of follow up.

Parameter	Study cohort^(a)^ (n = 188)	Change of T_50_ [%]		Sign.	Absolute T_50_		Sign.
		LOW (n = 94) < −22.7 %	HIGH (n = 94) > −22.7 %		LOW (n = 94) < 192.5 min	HIGH (n = 94) > 192.5 min	
**Age [y]**	71 [57–78]	73 [59–78]	67 [56–77]	0.20	72 [58–78]	69 [56–79]	0.65
**Gender [males]**	121 (64.4%)	57 (61%)	64 (68.1%)	0.36	59 (63%)	62 (66%)	0.76
**BMI [kg/m²]**	25.5 ± 5.7	26 ± 6.2	25 ± 5.1	0.04	26.1 ± 6.1	24.9 ± 5.3	0.09
**Adapted CCI [0–21]**	4 [2–7]	4 [2–6]	4 [1–7.3]	0.69	4 [2–6]	4 [1–7]	0.98
**History of MI**	21 (11.2%)	10 (11%)	11 (11.7%)	1	7 (7%)	14 (14.9%)	0.16
**Hypertension**	171 (91%)	87 (93%)	84 (89.4%)	0.61	84 (89%)	87 (92.6%)	0.61
**Diabetes**	75 (39.9%)	40 (43%)	35 (37.2%)	0.55	37 (39%)	38 (40.4%)	1
**Smoking [ever]**	83 (44.1%)	43 (46%)	40 (42.6%)	0.78	47 (50%)	36 (38.3%)	0.14
**Dyslipidaemia** ^*^	91 (48.4%)	45 (48%)	46 (48.9%)	1	45 (48%)	46 (48.9%)	1
**COPD**	21 (11.2%)	12 (13%)	9 (9.6%)	0.64	15 (16%)	6 (6.4%)	0.06
**Cancer**	45 (23.9%)	24 (26%)	21 (22.3%)	0.73	26 (28%)	19 (20.2%)	0.31
**CHD**	69 (36.7%)	34 (36%)	35 (37.2%)	1	33 (35%)	36 (38.3%)	0.76
**PAOD**	51 (27.1%)	30 (32%)	21 (22.3%)	0.19	24 (26%)	27 (28.7%)	0.74
**CerVD**	36 (19.1%)	21 (22%)	15 (16%)	0.35	20 (21%)	16 (17%)	0.60
**CHF**	35 (18.6%)	13 (14%)	22 (23.4%)	0.13	14 (15%)	21 (22.3%)	0.26
**Liver fibrosis**	12 (6.4%)	4 (4%)	8 (8.5%)	0.37	4 (4%)	8 (8.5%)	0.37
**GIT disease**	71 (37.8%)	38 (40%)	33 (35.1%)	0.55	43 (46%)	28 (29.8%)	0.04
**GIT bleeding**	28 (14.9%)	10 (11%)	18 (19.1%)	0.15	9 (10%)	19 (20.2%)	0.06
**Rheumatic dis**.	4 (2.1%)	3 (1.6%)	1 (0.5%)	0.62	4 (4%)	0 (0%)	0.12
**Dementia**	2 (1.1%)	1 (0.5%)	1 (0.5%)	1	2 (2%)	0 (0%)	0.50
**HD not HDF**	109 (87.2%)	52 (90%)	57 (85.1%)	0.59	63 (93%)	46 (80.7%)	0.06
**HD-vintage [mos.]**	62 [43–98]	62 [45–99]	60 [40–89]	0.26	58 [44–91]	63 [41–99]	0.85
**Kt/V**	1.5 ± 0.4	1.4 ± 0.4	1.5 ± 0.3	0.09 ^(a)^	1.4 ± 0.4	1.5 ± 0.3	0.03
**Catheter present**	10 (5.3%)	6 (6%)	4 (4.3%)	0.49	5 (5%)	5 (5.3%)	1
**Hgb [g/dl]**	11.5 ± 4.7	11.7 ± 6.5	11.3 ± 1.1	0.54	11.8 ± 6.5	11.2 ± 1.2	0.39
**Calcium [mmol]** ^**†**^	2.2 ± 0.2	2.2 ± 0.2	2.2 ± 0.2	0.82	2.2 ± 0.2	2.2 ± 0.2	0.79
**Δ calcium**	−0.1 ± 0.2	−0.1 ± 0.2	0 ± 0.2	0.21	−0.1 ± 0.2	−0.1 ± 0.2	0.95
**Magnesium [mmol]**	0.62 ± 0.11	0.62 ± 0.10	0.63 ± 0.12	0.49	0.61 ± 0.11	0.64 ± 0.12	0.15
**Phosphate [mmol]**	1.6 ± 0.5	1.7 ± 0.5	1.5 ± 0.4	<0.001	1.8 ± 0.5	1.4 ± 0.3	<0.001
**Δ phosphate**	−0.1 ± 0.5	0 ± 0.5	−0.2 ± 0.5	0.001	0 ± 0.5	−0.2 ± 0.4	0.07
**Albumin [g/l]**	38.9 ± 3.8	38.5 ± 4.1	39.4 ± 3.3	0.08	38.1 ± 3.9	39.8 ± 3.5	0.002
**Δ albumin [g/l]**	−1.4 ± 4.0	−1.8 ± 4.1	−1.3 ± 0.32	0.11	−1.5 ± 4.1	−1.3 ± 3.2	0.71
**Creatinine [mg/dl]**	8.6 ± 2.4	8.4 ± 2.1	8.7 ± 2.6	0.38	8.6 ± 2.3	8.5 ± 2.5	0.73
**BUN**	126.2 ± 33.7	125.3 ± 31.8	127.1 ± 35.5	0.72	127.4 ± 31.9	125 ± 35.4	0.62
**iPTH [pg/ml]** ^**‡**^	216 [106–402]	258 [124–450]	196 [86–360]	0.14	269 [141–424]	188 [77–319]	0.013
**IL6 [pg/ml]**	5.7 [0–12.8]	6.4 [0.8–12.2]	4.8 [0–13.1]	0.33	7.1 [0.8–13.5]	3.7 [0–10.5]	0.03
**Antihypertensives**	159 (84.6%)	83 (88%)	76 (80.9%)	0.23	82 (87%)	77 (81.9%)	0.42
**VDRA**	118 (62.8%)	62 (66%)	56 (59.6%)	0.45	60 (64%)	58 (61.7%)	0.88
**Phosphate binders**	147 (78.2%)	71 (37.8%)	76 (40.4%)	0.48	75 (80%)	72 (76.6%)	0.72
**- calcium cont**.	102 (54.3%)	48 (51%)	54 (57.4%)	0.24	41 (44%)	34 (36.2%)	0.46
**- others**	75 (39.9%)	36 (38%)	39 (41.5%)	0.76	41 (44%)	34 (36.2%)	0.37
**Cinacalcet**	52 (27,7%)	29 (30.9%)	23 (24.5)	0.42	29 (30.9)	23 (24.5%)	0.42
**Statins**	79 (42%)	42 (22.3%)	37 (19.7%)	0.56	38 (40%)	41 (43.6%)	0.78
**Marcumar**	29 (15.4%)	11 (5.9%)	18 (9.6%)	0.23	14 (15%)	15 (16%)	1

Age, comorbidities and basic laboratory values at time of 24 months FU; data is expressed as mean ± standard deviation (SD), median and [interquartile-range] or counts and (% of superset) for normally distributed data unpaired t-test, Mann-Whitney-U- and χ²-test were used for comparison; ^*^Dyslipidaemia: diagnosed (medical report) or intake of statins; ^†^two total calcium values were imputed as centres specific mean values. ^‡^12 missing values for intact parathyroid hormone (iPTH); Δ phosphate/Δ calcium/Δ albumin values were calculated as value^Follow up^−value^Baseline^. Abbreviations: Body mass index (BMI); Charlson Comorbidity Index (CCI); myocardial infarction (MI); chronic obstructive pulmonary disease (COPD); coronary heart disease (CHD); peripheral arterial occlusive disease (PAOD); cerebral vascular disease (CerVD); congestive heart failure (CHF); gastrointestinal (GIT); haemodialysis not haemodiafiltration (HD(F)); Interleukin-6 (IL-6); Vitamin D receptor activators (VDRA).

**Table 2:** Multivariable linear regression: dependent variable T_50_^Change^.

Parameter	1 SD	β - values	95 % confidence intervals of β	Standardized β - values	p - value
**Intercept**	—	−20.2	−29.6 to −10.9	—	0.001
**Age [years]**	14.4	−2.2	−6.5 to 2.2	−0.07	0.33
**Sex [male = 1]**		5.2	−3.6 to 13.9	0.08	0.24
**BMI [kg/m²]**	5.7	0.3	−4.1 to 4.7	0.01	0.88
**Calcium-containing phosphate binders =1**	—	7.0	−1.1 to 15.2	0.12	0.09
**VDRA intake =1**	—	−6.9	−15.4 to 1.7	−0.11	0.11
**CHD =1**	—	7.6	−2 to 17.2	0.12	0.12
**PAOD =1**	—	−7.0	−17.5 to 3.5	−0.1	0.19
**Diabetes =1**	—	−5.5	−14.3 to 3.4	−0.09	0.23
**Kt/V**	0.4	5.4	0.8 to 9.9	0.17	0.02
**Albumin [g/l]**	3.8	3.7	−1.3 to 8.7	0.12	0.15
**Δ albumin [g/l]**	4.0	2.1	−2.4 to 6.6	0.07	0.37
**Magnesium [mmol]**	0.1	4.0	−0.5 to 8.5	0.12	0.08
**calcium [mmol]**	0.2	1.7	−3.7 to 7.1	0.06	0.54
**Δ calcium [mmol]**	0.2	0.4	−4.7 to 5.5	0.01	0.88
**Phosphate [mmol]**	0.5	−6.9	−11.8 to −2.1	−0.23	**0.006**
**Δ phosphate [mmol]**	0.5	−8.4	−13.1 to −3.6	−0.27	**0.001**
**IL-6 [pg/ml]**	9.9	0.8	−3.5 to 5.1	0.03	0.727

Multivariable linear regression with T_50_^Change^ [%] as dependent variable. Corrected R^2^ of the model was 0.24. Variance of inflation factors were <2 for all variables. Metric variables were centred on their mean before entering the model. All variables were included at once. Patients characteristics at 24 months FU were used for modelling. Δ phosphate, Δ calcium and Δ albumin were calculated as value^Follow up^−value^Baseline^ Abbreviations: Body mass index (BMI); Vitamin D receptor activators (VDRA); coronary heart disease (CHD); peripheral arterial occlusive disease (PAOD); Interleukin-6 (IL-6).

### Declining T_50_ predicts all-cause and cardiovascular mortality in haemodialysis patients

**Figure 4:**
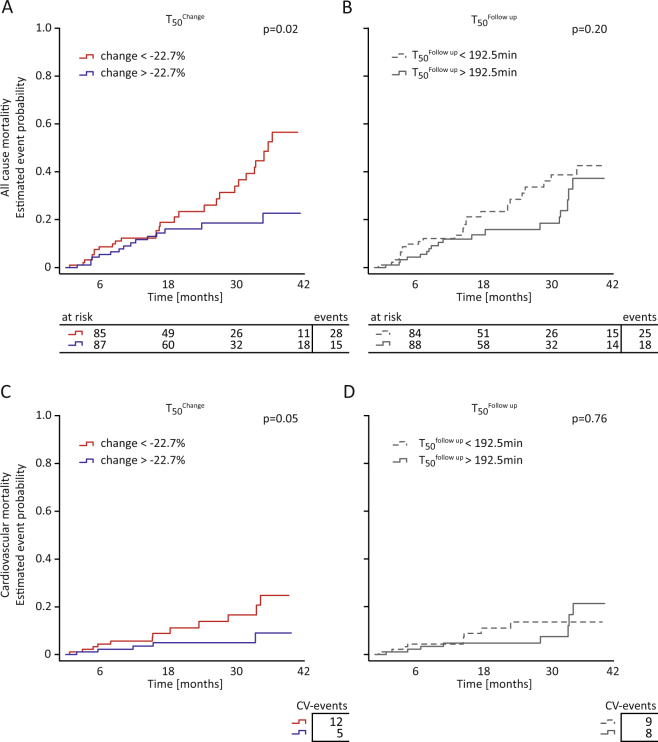
Univariate correlation of T_50_^Change^ and absolute T_50_^Change^. (**A**) Overall cumulative incidence functions stratified by median T_50_^Change^ (=−22.7%). (**B**) Overall cumulative incidence functions stratified by median T_50_^Follow up^ (=192.5 min). Patients at risk and total events per group are reported below the graphs. (**C**) Predicted cardiovascular death incidence functions stratified by median T_50_^Change^. (**D**) Predicted cardiovascular death incidence functions stratified by median T_50_^Follow up^. Number of lethal cardiovascular events are reported below the graphs. Log-rank statistics was used for comparison of incidence functions. p-values are reported in the upper right corner of each graph.

After 24 months of observation, patients were followed up for all-cause (n = 43) and CV mortality (n = 17) for a median of 19 [11–35] months. Three patients underwent renal transplantation and 12 were lost to FU (Fig. 1). In Kaplan Meier analysis, cumulative incidence of all-cause mortality was significantly higher in patients below the median of T_50_^Change^ (p = 0.02; Fig. 4A). Regarding CV-mortality a similar trend was visible although median T_50_^Change^ did not reach statistical significance (p = 0.05, Fig. 4C and D).

In contrast, stratification of patients by median of T_50_^Follow up^ didn’t significantly predict all-cause mortality in our cohort (Fig. 4B). Likewise, stratification of the study population by T_50_^Baseline^ was not significantly associated with all-cause mortality (Supplementary Figure 2), likely due to the preselection described above.

Considering T_50_^Change^ on a continuous scale, declining T_50_ was significantly associated with all-cause mortality in Cox-regression (HR = 1.74 per SD decline; 95%CI: 1,2 to 2,51; p = 0.003). After block-wise adjustment for demographics (age, sex, BMI), comorbidities and basic laboratory parameters, 1SD = 30.5% decline in T_50_ remained associated with a 51% (95%CI: 4–120%; p = 0.03) increased risk for all-cause mortality. Likewise, declining T_50_ was associated with CV mortality (HR = 2.14; 95%CI:1.15 to 3.97; Table 3). Exclusion of non-Caucasian study participants (n = 6) did not materially affect the latter results (supplementary Table 4).

**Table 3:** All-cause mortality: Crude and adjusted hazard ratios of T_50_^Change^ per SD decline (=30.5%).

Model	all-cause mortality		p - value	CV mortality		p - value
	HR per 1 SD decline	95 % CI		HR per 1 SD decline	95 % CI	
Crude T_50_^Change^	1.74	1.2 to 2.51	0.003	2.050	1.12 to 3.75	0.02
Model 1	1.71	1.17 to 2.51	0.006	2.140	1.15 to 3.97	0.02
Model 2	1.71	1.16 to 2.51	0.007	—	—	—
Model 3	1.61	1.09 to 2.36	0.02	—	—	—
Model 4^*^	1.51	1.04 to 2.2	0.03	—	—	—

Crude and adjusted hazard ratios (HR) are presented per 1SD decrease (=30.5%) of T50 during the 24 months follow up. All-cause mortality was the dependent variable. Model 1 includes age, sex, body mass index; Model 2 additionally includes the adapted Charlson Comorbidity Index; Model 3 additionally includes albumin; Model 4 additionally includes log transformed Interleukin-6; *addition of phosphate did not significantly improve the model.

By contrast, neither absolute T_50_^Follow up^ nor T_50_^Baseline^ remained significant predictors of all-cause mortality in the final adjusted model (supplementary Table 2 and supplementary Table 3, respectively) and were relevantly associated with CV mortality in our study (not shown).

## Discussion

The main findings of this subgroup analysis in chronic haemodialysis patients, were, firstly, that serum calcification propensity T_50_ declined between baseline and FU. Secondly, that a decline in T_50_ was accompanied by increasing phosphate levels at baseline and 24 months FU. Thirdly, that the intra-individual decline of calcification resistance predicts all-cause and cardiovascular mortality in HD patients. These findings suggest that observing change of T_50_ might add prognostic value to absolute T_50_ values in HD patients.

The development of the T_50_-test was an important step ahead for a more comprehensive assessment of colloidal chemical interactions within the complex field of “calcification and calcium x phosphate nanocrystal formation”^5,10^. Elevated serum phosphate, calcium, low magnesium and fetuin-A levels have long been linked to vascular calcification and mortality in ESRD and HD patients^14–25^. The colloidal chemical nature of the crystallization process per se suggests strong interaction effects amongst these players, which is in line with findings from cohort studies^16,26–28^. In fact, mortality is especially high for patients with both excessively high phosphate and calcium serum levels^21,28^. On the contrary, magnesium appears to dampen the detrimental effects of elevated phosphate levels in HD patients^16^. The strength of the T_50_-test is that it takes these interactions into account by assessing the distal tract of the calcification cascade, i.e. the interaction between calcium and phosphate, which takes place in the CPP-forming biological matrix of human serum^6^. Elevated serum phosphate, calcium and lower serum albumin have already been associated with lower absolute T_50_ at a single time point in CKD and HD patients^7,10^. Our data add further plausibility to this concept by indicating, that high or increasing phosphate, parallels declining T_50_. We did not, however, observe a similar relation for Δ albumin and Δ calcium, which might in part be due to a lower variance of these parameters between baseline and 24 months FU in our cohort. Nevertheless, our data underscore the importance of controlling serum phosphate levels in HD patients, as high or rising phosphate levels may well weaken the natural resistance against the formation of secondary CPP. However, given the moderate fit of our linear regression model, besides Δ phosphate or Kt/V other factors will also relate to declining T_50_. Magnesium is such a candidate indicated by our data. Unfortunately, we were unable to investigate Δ ionized magnesium levels over time, due to an incomplete magnesium-dataset at baseline.

Given that a short T_50_ time represents lower resistance to secondary CPP formation^5,6^, a decline of T_50_, as observed in our study, implies the loss of anti-calcific capacities of biologic fluids. In our cohort, a decline of T_50_ occurred over the course of dialysis dependency and was independently associated with all-cause mortality and remained associated with cardiovascular mortality after adjustment for potential confounders. As such, the per cent decline of T_50_ between baseline and 24-months reassessment showed a considerably stronger association with mortality than absolute T_50_^Follow up^. Since absolute T_50_ values are to a large extent genetically determined in the general population^13^, it appears plausible that the dynamics of T_50_ predict future risk more accurately in HD patient, which face longitudinal weakening of their “natural“ calcification resistance. By analogy, prognosis of e.g. calcific aortic stenosis is not only determined by the absolute reduction of valve area but also the rapidity of stenosis progression which is especially high in HD patients^29,30^. Based on this reasoning, it is tempting to speculate that preventing a decline of T_50_ in HD patients or restoring T_50_ in those who present with declining T_50_ might favourably affect the future clinical course. Importantly, T_50_ represented the only modifiable risk factor in our adjusted Cox model for mortality. In fact, first interventional pilot studies suggest that increasing dialysate magnesium and bicarbonate act concordantly to prolong T_50_ time in HD patients^31^. Similarly, phosphate binder therapy has the potential to elevate T_50_^32^.

Our study has several limitations. Although this is the first study to assess individual dynamics of serum calcification propensity in HD patients it doesn’t fulfil all criteria for studying change, since T_50_ was only assessed from sera collected at two not three different time points. In addition, the study design required exclusion of patients who did not survive the first 24 months FU period or who missed timely FU. Therefore, the study population was significantly enriched for survivors, which limits the generalizability of our results and explains the lack of predictive potential of T_50_^Baseline^. Due to the relatively low number of cardiovascular deaths we were unable to fully adjust the Cox model for cardiovascular mortality. The study design and post hoc character of the study preclude conclusions regarding causality and therefore, the pathophysiologic mechanism linking declining T_50_ to excess mortality remains elusive. We could not consider dietary calcium or phosphorus intake since these parameters were not available in the dataset. Lastly, although these data provide a fascinating new perspective on intra-individual serum calcification resistance and the interpretation of the T_50_ test, larger scaled studies are needed to replicate and develop these results.

In conclusion, longitudinally monitoring changes in T_50_-time in HD patients might offer a suitable tool to identify patients at risk for adverse outcome and open the conceptual possibility of basing multimodal and personalized therapeutic interventions on longitudinal changes of T_50_^5^. Larger scaled prospective interventional studies are needed to put this concept to a test.

## Methods

### Subjects/Study population

This study was undertaken in a subset of patients of the original “rISk strAtification in end stage Renal disease” - (ISAR)-cohort, a multicentre, prospective longitudinal observatory cohort study^33^. Between 2010 and 2013, 519 stable HD patients from 17 dialysis centres in Munich, Germany and the surrounding area were included. Out of these patients the present study included 188 patients, which were still alive and active in the study after the first FU period of 24 months with a maximum spread of ±3.2 months and for whom sera were available at both baseline and follow-up. Patients for whom timely 24 [23–25] months FU had been missed were excluded a priori (Fig. 1). Patients were ≥18 years of age, had an HD vintage of least 90 days and gave written and informed consent. The ISAR study was approved by the ethics committees of the Klinikum rechts der Isar, Technical University Munich and of the Bavarian State Board of Physicians^33^. It was carried out in accordance with the declaration of Helsinki. Written informed consent was obtained from all participants. The study was registered under ClinicalTrials.gov identifier: NCT01152892 prior to its start. For the derived study, no additional ethics committee approval was sought. For more details, we refer to the study protocol^33^.

### Clinical data assessment

Patients’ age, comorbidities and medication at FU were assessed using medical records from the contributing dialysis centres. Comorbidities were recorded following an adapted version of the Charlson Comorbidity Index (CCI) for ESRD as introduced by Liu *et al*.^34^. BMI at FU was calculated as body weight/height^2^ [kg/m^2^]. Access type (fistula or permanent catheter) was determined at time of FU and considered constant for the consecutive observation period. Information on dialysis prescription (Ultrafiltration, session duration, Kt/V as a measure of dialysis efficiency, HD/HDF, anticoagulation) was provided by the contributing centres. All patients underwent regular bicarbonate dialysis with synthetic membranes. For details, we refer to the study protocol^33^.

### Endpoints

The primary outcome parameters for the ISAR-trial and this project were all-cause and CV mortality. Observation for all-cause and cause-specific mortality started the day after the 24 month FU visit. In the absence of a final medical report stating the cause of death, attending physicians and relatives were contacted to confirm death and gather available information, based on which the ISAR-study physician board assigned each case an underlying cause of death. CV mortality (n = 17) was due to sudden cardiac death (n = 6), myocardial infarction (n = 4), heart failure (n = 1), cardiac surgical procedure (n = 2), pulmonary embolism (n = 1), major stroke (n = 2) and rupture of aortic aneurysm (n = 1). Non-CV lethal events (n = 26) comprised infectious events, gastrointestinal bleeding, non-CV related surgery, withdrawal from treatment, chronic pulmonary disease, malignant diseases and unknown causes of death.

### Blood specimen collection and Laboratory methods

Serum was collected prior to a midweek dialysis session at time of inclusion and at FU. Serum was centrifuged after 30 min of resting at room temperature, aliquoted and frozen at −20 °C, transferred to our laboratory on dry ice and stored at −80 °C for later analysis. Routine laboratory analysis was performed by ISO certified laboratories. IL-6 was determined using BD Flex-sets on a FACS Canto II and BD Diva software following the manufacturer’s instructions. BD FCAP Array software 3.0 was used for analysis. Ionized magnesium levels were determined from frozen sera using the Nova 8 Analyzer (Nova Biomedical, Waltham, MA, US). T_50_ was determined at previously described by Pasch *et al*.^6,10^. Sera had never been thawed prior to analysis and were sent to Bern on dry ice and analyzed in a blinded manner. Median storage duration was 48 [42–70] or 24 [18–47] months for samples collected at baseline or 24 months follow, respectively.

### Statistical Analysis

Statistical analysis was performed using IBM SPSS Statistics 23 and R version 3.3.2 software. We present mean ± standard deviation (SD), median and [interquartile-range] or counts and (% of superset) for normally distributed data, non-normally distributed metric and ordinal variables or nominal data, respectively. IL-6 levels were natural logarithm-(ln)-transformed to adjust for skewness of distribution.

Paired t-test was used to compare differences between absolute T_50_-values at baseline and 24 months FU. Change in T_50_ (T_50_^Change^) was calculated as (T_50_^Follow up^−T_50_^Baseline^)/T_50_^Baseline^ and expressed in percent. Subjects were stratified above and below the median of T_50_^Change^ and T_50_^Follow up^, respectively for description of baseline characteristics at time of 24 months FU. Group differences were tested using unpaired t-test, Mann-Whitney-U, χ²-test and paired t-test as appropriate. Additionally, we assessed associations of continuous variables with T_50_^Change^ and T_50_^Follow up^ using Pearson correlation. Median T_50_^Follow up^ and median T_50_^Change^ were also used to stratify patients for univariate Kaplan-Meier survival analysis of all-cause and CV mortality. Cumulative incidence functions were estimated for CV and non-CV deaths to account for competing risks. Log-rank tests were performed to compare (cause specific) hazard rates between relevant groups. Patients were censored at time of transplantation or at time of loss to FU.

A multivariable linear regression model was built including age, sex, BMI, coronary heart disease (CHD), peripheral arterial occlusive disease (PAOD), diabetes, hypertension, intake of calcium containing phosphate binders, Vitamin D3 supplementation and laboratory parameter (calcium, magnesium, albumin, phosphate, intra-individual Δ-phosphate (=phosphate^Follow up^−phosphate^Baseline^ [mmol/l]) and Δ-calcium, Δ-albumin to identify factors that were associated with T_50_^Change^. Coefficients of regression are reported per 1 SD increase of the regressor. Variance of inflation factors were below 2 for all entered variables. Cox proportional hazard models were fit to the data to characterize the association of T_50_^Change^ with all-cause mortality. Proportionality of hazard rates was investigated by the use of Schoenfeld residuals and a statistical test for proportional hazard proposed by Grambsch and Therneau^35^.

The models were adjusted for demographic factors (age, gender, BMI), comorbidities (following the adapted CCI as proposed by Liu *et al*.^34^), albumin and ln-transformed IL-6. Hazard ratios (HR) and 95 % confidence interval (95% CI) are reported per 1SD of T_50_^Change^ (≈30.5 % change in T_50_) during the 24 months FU, 2 of 188 cases were censored before the earliest event had occurred. The datasets generated during and/or analysed during the current study are available from the corresponding author on reasonable request.

## Electronic supplementary material

Worsening calcification propensity precedes all-cause and cardiovascular mortality in haemodialyzed patients

## References

[1] Go AS, Chertow GM, Fan D, McCulloch CE, Hsu C-y (2004). Chronic Kidney Disease and the Risks of Death, Cardiovascular Events, and Hospitalization. N. Engl. J. Med..

[2] Foley RN, Parfrey PS, Sarnak MJ (1998). Epidemiology of cardiovascular disease in chronic renal disease. J. Am. Soc. Nephrol..

[3] Stokes JB (2011). Consequences of Frequent Hemodialysis: Comparison to Conventional Hemodialysis and Transplantation. Trans. Am. Clin. Climatol. Assoc..

[4] Mizobuchi M, Towler D, Slatopolsky E (2009). Vascular calcification: the killer of patients with chronic kidney disease. J. Am. Soc. Nephrol..

[5] Pasch A (2016). Novel assessments of systemic calcification propensity. Curr. Opin. Nephrol. Hypertens..

[6] Pasch A (2012). Nanoparticle-based test measures overall propensity for calcification in serum. J. Am. Soc. Nephrol..

[7] Smith ER (2014). Serum calcification propensity predicts all-cause mortality in predialysis CKD. J. Am. Soc. Nephrol..

[8] Dahle DO (2016). Serum Calcification Propensity Is a Strong and Independent Determinant of Cardiac and All-Cause Mortality in Kidney Transplant Recipients. Am. J. Transplant..

[9] Keyzer CA (2016). Calcification Propensity and Survival among Renal Transplant Recipients. J. Am. Soc. Nephrol..

[10] Pasch A (2017). Blood Calcification Propensity, Cardiovascular Events, and Survival in Patients Receiving Hemodialysis in the EVOLVE Trial. Clin. J. Am. Soc. Nephrol..

[11] Chertow GM (2012). Effect of cinacalcet on cardiovascular disease in patients undergoing dialysis. N. Engl. J. Med..

[12] Shroff RC (2008). Dialysis accelerates medial vascular calcification in part by triggering smooth muscle cell apoptosis. Circulation.

[13] Edward Pivin, M. B., Devuyst, O., Huynh-Do, U. & Bochud, M. Andreas Pasch Serum Calcification Propensity Is Largely Genetically Determined in the General Population [abstract]. *ASN Kidney Week 2016* FR-PO423 (2016).

[14] Yu L, Li H, Wang SX (2017). Serum Magnesium and Mortality in Maintenance Hemodialysis Patients. Blood Purif..

[15] Hermans MM (2007). Association of serum fetuin-A levels with mortality in dialysis patients. Kidney Int..

[16] Sakaguchi Y, Hamano T, Isaka Y (2017). Effects of Magnesium on the Phosphate Toxicity in Chronic Kidney Disease: Time for Intervention Studies. Nutrients.

[17] Tentori F (2008). Mortality risk for dialysis patients with different levels of serum calcium, phosphorus, and PTH: the Dialysis Outcomes and Practice Patterns Study (DOPPS). Am. J. Kidney Dis..

[18] Westenfeld R (2009). Fetuin-A Protects against Atherosclerotic Calcification in CKD. Journal of the American Society of Nephrology: JASN.

[19] Zhang K (2013). Malnutrition, a new inducer for arterial calcification in hemodialysis patients?. J. Transl. Med..

[20] Posadas-Sanchez R (2016). Serum magnesium is inversely associated with coronary artery calcification in the Genetics of Atherosclerotic Disease (GEA) study. Nutr. J..

[21] Melamed ML (2006). Changes in serum calcium, phosphate, and PTH and the risk of death in incident dialysis patients: a longitudinal study. Kidney Int..

[22] Dautova Y (2014). Fetuin-A and Albumin Alter Cytotoxic Effects of Calcium Phosphate Nanoparticles on Human Vascular Smooth Muscle Cells. PLoS One.

[23] Ishimura E (2007). Significant association between the presence of peripheral vascular calcification and lower serum magnesium in hemodialysis patients. Clin. Nephrol..

[24] Meema HE, Oreopoulos DG, Rapoport A (1987). Serum magnesium level and arterial calcification in end-stage renal disease. Kidney Int..

[25] Isakova T (2009). Phosphorus Binders and Survival on Hemodialysis. Journal of the American Society of Nephrology: JASN.

[26] Sakaguchi Y (2016). Association between Density of Coronary Artery Calcification and Serum Magnesium Levels among Patients with Chronic Kidney Disease. PLoS One.

[27] Jahromi MT, Yao G, Cerruti M (2013). The importance of amino acid interactions in the crystallization of hydroxyapatite. Journal of the Royal Society Interface.

[28] Stevens LA, Djurdjev O, Cardew S, Cameron EC, Levin A (2004). Calcium, phosphate, and parathyroid hormone levels in combination and as a function of dialysis duration predict mortality: evidence for the complexity of the association between mineral metabolism and outcomes. J. Am. Soc. Nephrol..

[29] Perkovic, V., Hunt, D., Griffin, S. V., du Plessis, M. & Becker, G. J. Accelerated progression of calcific aortic stenosis in dialysis patients. *Nephron Clin. Pract*. **94**, c40–45, doi:71280 (2003). 10.1159/000071280 12845236

[30] Kamath AR, Pai RG (2008). Risk factors for progression of calcific aortic stenosis and potential therapeutic targets. The International Journal of Angiology: Official Publication of the International College of Angiology, Inc.

[31] Andreas Pasch, M. B., Edward, R. S., Benackova, K. & Uehlinger, D. E. Serum Calcification Propensity Is Improved by Increased Dialysate Bicarbonate and Dialysate Magnesium: The BicMag Pilot Study [abstract]. *ASN Kindey Week 2016* FR-PO401 (2016).

[32] Pasch Andreas, D. F. A. L. M., Adrian, C., Barbara, M., Arens Hans, J. & Passlick-Deetjen Jutta, J-D. W. Serum calcification propensity of HD patients is therapeutically improved by a calcium acetate/magnesium carbonate containing phosphate binder [abstract]. *51st Congress of the European-Renal-Association*(*ERA*)/*European-Dialysis-and-Transplant-Association* (*EDTA*)*, May 31-Jun 03, 2014, Amsterdam, Netherlands* (2014).

[33] Schmaderer C (2016). Rationale and study design of the prospective, longitudinal, observational cohort study "rISk strAtification in end-stage renal disease" (ISAR) study. BMC Nephrol..

[34] Liu J, Huang Z, Gilbertson DT, Foley RN, Collins AJ (2010). An improved comorbidity index for outcome analyses among dialysis patients. Kidney Int..

[35] Grambsch PM, Therneau TM (1994). Proportional hazards tests and diagnostics based on weighted residuals. Biometrika.

