# Supplementary material for: Worsening calcification propensity precedes all-cause and cardiovascular mortality in haemodialyzed patients
Source: Sci Rep. 2017 Oct 17;7:13368. doi: 10.1038/s41598-017-12859-6 (PMC5645333; doi:10.1038/s41598-017-12859-6)
Supplement: Supplementary file 1 — Worsening calcification propensity precedes all-cause and cardiovascular mortality in haemodialyzed patients [file 41598_2017_12859_MOESM1_ESM.doc]

**Title: “Worsening calcification propensity precedes all-cause and cardiovascular mortality in haemodialyzed patients**”

Dr. Georg Lorenz^1*†^ and Dr. Dominik Steubl^1†^, Dr. Stephan Kemmner^1^, PD. Dr. Andreas Pasch^2^, Wilhelm Koch-Sembdner^1^, Dang Pham^1^, Dr. Bernhard Haller^3^, Quirin Bachmann^1^, Dr. Christopher C. Mayer ^4^, Dr. Siegfried Wassertheurer ^4^, Dr. Susanne Angermann^1^, PD. Dr. Maciej Lech^1^, Dr. Philipp Moog^1^, Prof. Dr. Axel Bauer ^5^, Prof. Dr. Uwe Heemann^1^, PD. Dr. Christoph Schmaderer^1*^

^1^ Department of nephrology, Klinikum rechts der ISAR, Technical University Munich, Germany

^2^ Calciscon AG, Biel-Nidau, Switzerland

^3^ Institute of Medical Statistics and Epidemiology, Technical University Munich, Germany

^4^ AIT Austrian Institute of Technology, Center for Health & Bioresources, Biomedical Systems, Vienna, Austria

^5^ Department of cardiology Ludwig-Maximilian University, Munich, Germany

* these authors are corresponding authors

† these authors contributed equally

Table of contents:

Supplementary table 1 (S-2)

Supplementary table 2 (S-3)

Supplementary table 3 (S-4)

Supplementary table 4 (S-5)

Supplementary figure 1 (S-6)

Supplementary figure 2 (S-7)

**Supplementary Table 1:** Study population versus total cohort at baseline

| **Parameter** | **ISAR cohort**  (n=515) |  | **Study population**  (n=188) |  |
| --- | --- | --- | --- | --- |
|  | mean ± SD; median [IQR]; count (% of superset) |  | mean ± SD; median [IQR]; count (% of superset) | Sign. |
| **Age [y]** | 67.9 [53.9-77.2] |  | 68.5 [55.3-75.7] | 0.93 |
| **Gender [males]** | 358 (69%) |  | 121 (64.4%) | 0.09 |
| **BMI [kg/m²]** | 26.3 ± 5.5 |  | 26.3 ± 5.7 | 0.57 |
|  |  |  |  |  |
| **Adapted CCI [0-21]** | 3 [1-6] |  | 3 [1-6] | 0.26 |
| **CHD** | 183 (35.3%) |  | 59 (32.2%) | 0.18 |
| **PAOD** | 123 (23.7%) |  | 44 (23.4%) | 1 |
| **History of MI** | 103 (19.8%) |  | 27 (14.4%) | 0.02 |
| **Hypertension** | 486 (93.6%) |  | 171 (90.9%) | 0.06 |
| **Diabetes** | 209 (40.3%) |  | 73 (38.8%) | 0.64 |
| **Smoking [ever]** | 118 (22.7%) |  | 49 (26.1%) | <0.001 |
|  |  |  |  |  |
| **Creatinine [mg/dl]** | 8.5 ± 2.7 |  | 8.4 ± 2.6 | 0.70 |
| **Phosphate [mmol]** | 1.7 ± 0.5 |  | 1.7 ± 0.5 | 0.89 |
| **Haemoglobin [g/dl]** | 11.8 ± 1.2 |  | 11.8 ± 1.1 | 0.44 |
| **Total Calcium[mmol] ^(*)^** | 2.26 ± 0.2 |  | 2.29 ± 0.2 | 0.005 |
| **Albumin [g/l]** | 40 ± 4 |  | 41 ± 4 | 0.04 |
|  |  |  |  |  |
| **HD(F)** | 444 (85.5%) |  | 157 (83.5%) | 0.36 |
| **HD vintage [months]** | 40.3 [20.6-71.2] |  | 36.6 [19.7-73.6] | 0.46 |
| **Ultrafiltration [l]** | 2.3 [1.5-3.0] |  | 2.2 [1.4-3.0] | 0.82 |
| **Kt/V** | 1.5 ± 0.4 |  | 1.5 ± 0.4 | 0.86 |
| **Catheter present** | 33 (7.3%) |  | 5 (2.7%) | 0.001 |
|  |  |  |  |  |
| **All-cause mortality** | 186 (35.8%) |  | 43 (22.9%) | <0.001 |

Data are reported as mean ± standard deviation or median and [interquartile-range] according to the distribution of metric data and for ordinal variables. For nominal parameters, we report counts and (% of superset). Right column: Differences in distributions were compared between in- and excluded patients at time of baseline using unpaired t-test, Mann-Whitney-U- and χ²-test as appropriate. * Two total calcium values were imputed by centre specific mean values. Abbreviations: Body mass index (BMI); Charlson Comorbidity Index (CCI); coronary heart disease (CHD); peripheral arterial occlusive disease (PAOD); myocardial infarction (MI); haemodialysis not haemodiafiltration (HD(F)); Interleukin-6 (IL-6); Vitamin D receptor activators (VDRA).

**Supplementary table 2:** T_50_^follow up^ entered to the adjusted Cox model 4 (all-cause mortality):

| **T_50_^Follow up^** | **1 SD** | **HR per SD increase** | **95 % confidence intervals** | **p - value** |
| --- | --- | --- | --- | --- |
| **T_50_^follow up^ [min]** | 67.8 | 0.82 | 0.59 to 1.15 | 0.26 |
| **Age [y]** | 14.4 | 1.60 | 1.08 to 2.37 | 0.02 |
| **Sex [female]** | - | 0.95 | 0.5 to 1.81 | 0.88 |
| **BMI [kg/m²]** | 5.7 | 0.97 | 0.7 to 1.35 | 0.86 |
| **Adapted CCI** | - | 1.22 | 1.12 to 1.34 | <0.001 |
| **Albumin [g/l]** | 3.7 | 0.68 | 0.50 to 0.92 | 0.01 |
| **Ln IL-6** | 1.2 | 1.62 | 1.08 to 2.44 | 0.02 |

T_50_^follow up^ entered the adjusted Cox model 4 (see main text). Abbreviations: Body mass index (BMI); natural-logarithm transformed interleukin-6 (ln IL-6). Hazard ratios (HR) are expressed per 1 standard deviation (SD) increase of the independent variables.

**Supplementary table 3: Crude and adjusted HR per 1SD of absolute T_50_^Baseline^ (all-cause mortality):**

| **T_50_^Baseline^** | **HR per 1 SD increase (=63.6 min)** | **95 % confidence intervals** | **p - value** |
| --- | --- | --- | --- |
| **Crude all-cause mortality** | 1.04 | 0.76 to 1.41 | 0.81 |
| **Adjusted model 4** | 1.15 | 0.85 to 1.54 | 0.37 |
| **Adjusted model 4* (using basal data)** | 1.03 | 0.76 to 1.40 | 0.84 |

T_50_^Baseline^ entered the crude and the adjusted Cox model 4 (adjusted for age, sex, body mass index, the adjusted CCI, serum albumin and logarithm transformed interleukin-6 at time of follow up - see main text). Similar results were obtained after addition of T_50_^Baseline^, when the model was adjusted for those parameters at baseline (adjusted model 4*). Four IL-6 values were missing at baseline.

**Supplementary table 4:** Sensitivity analysis: Exclusion of 6 patients: of African (n=4; 2%) or Asian (n=2, 1%) ethnical origin.

| **T_50_^Change^**  **^↓^** | **Outcome** | **Study Population (n=188)** | |  | **Exclusion of non-Caucasians (n^RMNG^ =182)** | |  |
| --- | --- | --- | --- | --- | --- | --- | --- |
|  |  | HR per 1 SD decline [95 % CI] | | p value | HR per 1 SD decline [95 % CI] | | p value |
| **Crude** | All-cause mortality | 1.74 [1.2 to 2.51] | | 0.003 | 1.72 [1.19 to 2.48 | | 0.004 |
| **Model 1** | All-cause mortality | 1.71 [1.17 to 2.51] | | 0.006 | 1.69 [1.15 to 2.48] | | 0.007 |
| **Model 2** | All-cause mortality | 1.71 [1.16 to 2.51] | | 0.007 | 1.66 [1.14 to 2.42] | | 0.008 |
| **Model 3** | All-cause mortality | 1.61 [1.09 to 2.36] | | 0.02 | 1.57 [1.07 to 2.29] | | 0.02 |
| **Model 4** | All-cause mortality | 1.51 [1.04 to 2.2] | | 0.03 | 1.49 [1.02 to 2.17] | | 0.04 |
|  |  |  |  |  |  |  |  |
| **Crude** | CV mortality | 2.05 [1.12 to 3.75] | | 0.02 | 2.03 [1.1 to 3.76] | | 0.02 |
| **Model 1** | CV mortality | 2.14 [1.15 to 3.97] | | 0.03 | 2.08 [1.1 to 3.89] | | 0.02 |

Crude and adjusted hazard ratios (HR) are presented per 1SD decrease (=30.5%) of T_50_^Change^ during the 24 months follow up. All-cause mortality was the dependent variable. Model 1 includes age, sex, body mass index; Model 2 additionally includes the adapted Charlson Comorbidity Index; Model 3 additionally includes albumin; Model 4 additionally includes log transformed Interleukin-6; Outcomes were all-cause and cardiovascular mortality as indicated in the second column. The third column reflects hazard ratios for T_50_^Change^ when all patients including 4 African and 2 Asian participants were included. The fourth column reports similar results, when these patients were excluded from analyses. Abbreviations: CV: cardiovascular, RMNG= remaining.

**Supplementary figure 1:** A: Histogram of absolute T_50_^baseline^ B: Histogram of absolute values of T_50_^follow up^; normal distribution curves were overlaid; Kolmogorow-Smirnow test was used to test for deviation from normality. p-values are displayed in the upper right corner of each histogram.

**
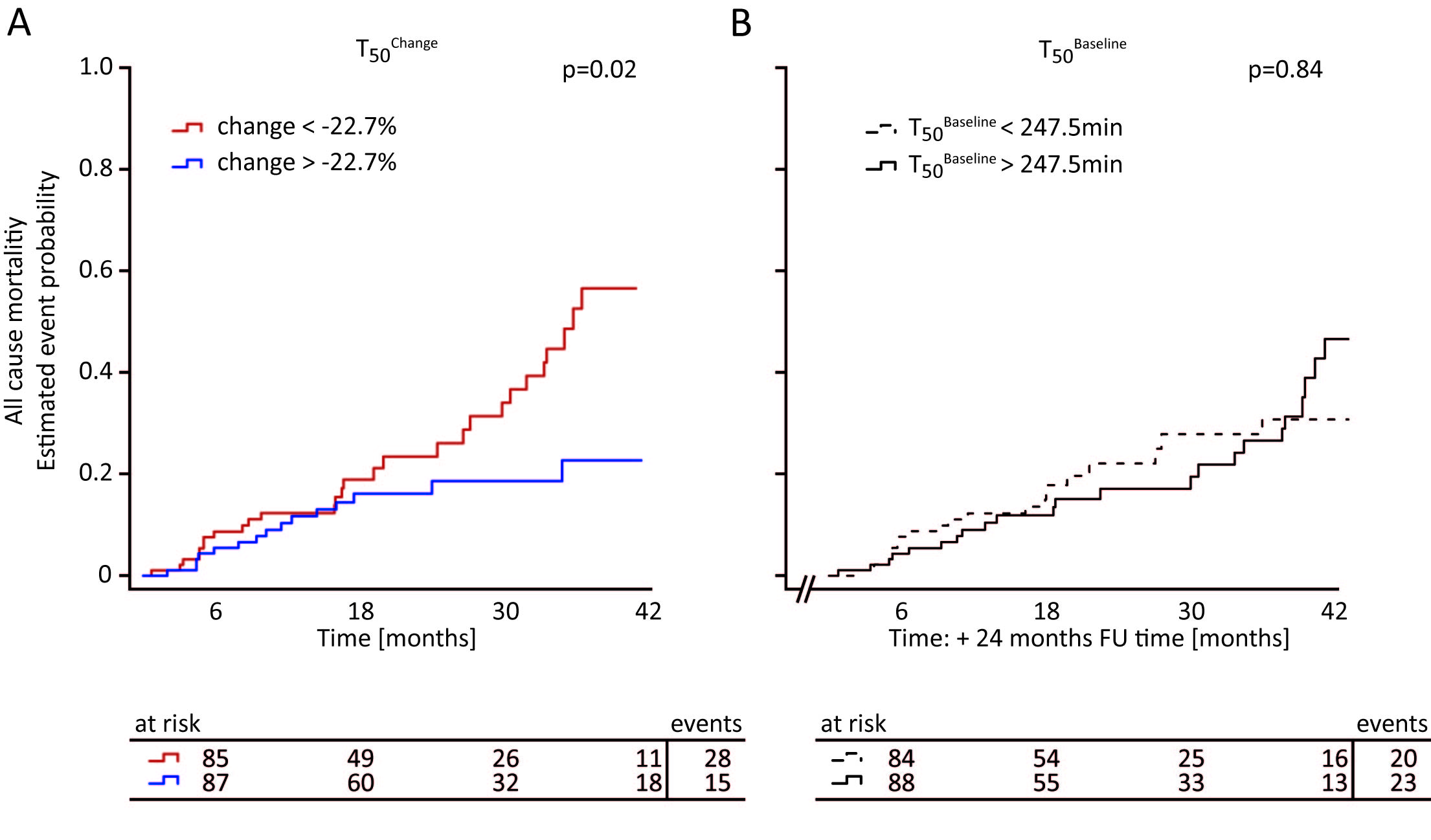
**

**Supplementary figure 2:** Cumulative incidence functions for all-cause mortality using basal T_50_ data**.** A: Overall cumulative incidence functions stratified by median T_50_^Change^ (=-22.7%) – identical to figure 4A (main text). B: Overall cumulative incidence functions stratified by median T_50_^Baseline^ (=247.5min). The period between inclusion and 24 months follow up (no events due to the study design) was truncated. Patients at risk and total events per group are reported below the graphs. Log-rank statistics was used for comparison of incidence functions. p-values are reported in the upper right corner of each graph. T_50_^Baseline^ did not show a relevant association with all-cause mortality in our study population.
